# Hepatitis C Virus Infection: Host–Virus Interaction and Mechanisms of Viral Persistence

**DOI:** 10.3390/cells8040376

**Published:** 2019-04-25

**Authors:** DeGaulle I. Chigbu, Ronak Loonawat, Mohit Sehgal, Dip Patel, Pooja Jain

**Affiliations:** 1 Department of Microbiology and Immunology, and the Institute for Molecular Medicine and Infectious Disease, Drexel University College of Medicine, 2900 West Queen Lane, Philadelphia, PA 19129, USA; dic26@drexel.edu (D.I.C.); rsl64@drexel.edu (R.L.); dp867@drexel.edu (D.P.); 2 Pennsylvania College of Optometry at Salus University, Elkins Park, PA 19027, USA; 3 Immunology, Microenvironment & Metastasis Program, The Wistar Institute, Philadelphia, PA 19104, USA; msehgal@wistar.org

**Keywords:** HCV, immune dysregulation, viral persistence, dendritic cells, interferons, T cells, NK cells

## Abstract

Hepatitis C (HCV) is a major cause of liver disease, in which a third of individuals with chronic HCV infections may develop liver cirrhosis. In a chronic HCV infection, host immune factors along with the actions of HCV proteins that promote viral persistence and dysregulation of the immune system have an impact on immunopathogenesis of HCV-induced hepatitis. The genome of HCV encodes a single polyprotein, which is translated and processed into structural and nonstructural proteins. These HCV proteins are the target of the innate and adaptive immune system of the host. Retinoic acid-inducible gene-I (RIG-I)-like receptors and Toll-like receptors are the main pattern recognition receptors that recognize HCV pathogen-associated molecular patterns. This interaction results in a downstream cascade that generates antiviral cytokines including interferons. The cytolysis of HCV-infected hepatocytes is mediated by perforin and granzyme B secreted by cytotoxic T lymphocyte (CTL) and natural killer (NK) cells, whereas noncytolytic HCV clearance is mediated by interferon gamma (IFN-γ) secreted by CTL and NK cells. A host–HCV interaction determines whether the acute phase of an HCV infection will undergo complete resolution or progress to the development of viral persistence with a consequential progression to chronic HCV infection. Furthermore, these host–HCV interactions could pose a challenge to developing an HCV vaccine. This review will focus on the role of the innate and adaptive immunity in HCV infection, the failure of the immune response to clear an HCV infection, and the factors that promote viral persistence.

## 1. Introduction

The liver is the metabolism hub of the body, which is responsible for all major anabolic and catabolic activities for survival. Hepatocarcinogenesis and inflammation cause liver damage, which, in turn, affects the functional efficiency of the liver [1]. Viruses such as hepatotropic viruses cause chronic infection of the liver, in which there is a gradual transformation of virally infected hepatocytes [2]. Hepatitis C (HCV) is one of the most dangerous and potent hepatotropic viruses that cause human infection. HCV causes an inflammation of the liver; however, a chronic HCV infection can lead to liver failure, liver cirrhosis, and hepatocellular carcinoma (HCC). 

Hepatitis C viruses do not differentiate between continental boundaries and, thus, can be found in almost all places of human habitation. For the Hepatitis B Virus, the western Pacific and African regions make up a larger bias for the prevalence by contributing 6.2% and 6.1% of the total infections respectively [3], whereas for Hepatitis C, the Eastern Mediterranean and European regions take major shares of 2.3% and 1.5% respectively [4]. The number of infected people with HCV are relatively higher than most viruses and that is one of the reasons why HCV is considered clinically very important. According to WHO, the number of chronically infected patients with HCV is 71 million worldwide [3,4]. The terminology of acute and chronic infections is a major criterion to classify the type and severity of the infection. An acute infection means the body is able to clear the virus within 6 months of incidence, whereas in a chronic infection, the immune system is unable to nullify the threat and the virus is persistent [5]. Some of these infections are coinfections with different viruses such as HIV, which furthers the complication [6,7]. The number of deaths attributed to chronic infections with HCV is astonishing, which is corroborated by reports of 399,000 deaths each year due to HCV-related liver inflammation [8]. HCV is mainly transmitted via exposure to HCV-infected blood and bodily fluids. The neonatal exposure to infected mothers, solid organ transplantations, unprotected sexual contact, and intravenous drug use are risk factors for HCV transmission [9]. HCV is an overloading economic burden for the healthcare system, and hence, actual expenditure statistics are monumental. For HCV, around $300 million is spent on liver transplant every year and the economic burden for infected patient healthcare costs approaches $9 billion [10]. All these reasons make up a powerful stimulus to study and eradicate these viruses.

The inability of the immune system to eliminate pathogens often results in the development of a persistent viral infection. A persistent HCV infection leads to chronic hepatitis and eventually causes cirrhosis and hepatocellular carcinoma. HCV persistence in the host can be attributed to the ability of the virus to evade immune surveillance by means such as viral mutation and an inhibition of innate immune cells such as dendritic cell (DC) and natural killer (NK) cells by HCV viral proteins, as well as by an alteration of the innate and adaptive arms of the immune response. This review focuses on the interactions of HCV with the host immune system and the mechanisms responsible for the development of viral persistence and subsequent progression to a chronic HCV infection.

## 2. Characteristics, Structure, and Pathogenesis

Discovered in 1989 at Chiron, HCV is a member of the Flaviviridae family, which hosts members like Dengue virus and Zika virus [11]. It has a single-stranded RNA genome under a lipid bilayer envelope that consists of approximately 9.5-kb genomes encoding a single open reading frame (ORF) that, when translated, results in the production of an approximately 3000-amino-acid-long polyprotein [12]. The polyprotein is subsequently translated and processed into three structural (core (C), envelope 1 (E1), and envelope 2 (E2)) and seven nonstructural proteins (viroporin p7, nonstructural proteins 2 (NS2), NS3, NS4A, NS4B, NS5A, and NS5B) [13,14]. The nonstructural proteins have critical functions in viral replication. NS3 has a C-terminal region that contains the RNA helicase and nucleoside triphosphate (NTPase) and a N-terminal region containing the NS serine protease [15]. NS5B is another important enzyme involved in viral transcription and replication, as well as an RNA-dependent RNA polymerase of HCV [15]. Several viral proteins also appear to play a role in the evasion of host immune responses. In addition to infecting hepatocytes, HCV has been reported to infect dendritic cells (DCs) [16,17], B cells [18,19], and peripheral blood mononuclear cells [20,21]. NS3, NS4A, NS4B, NS5A, and NS5B forms the replicase machinery and NS2 and p7 are essential for viral assembly and release [22]. HCV-associated pathogenesis may be attributed to the type of genotype causing an infection of the liver in the host. For example, the Type 1 genotype is more aggressive and more directly linked to HCC and cirrhosis, and Type 3 is associated with steatosis and fibrosis [23,24]. Additionally, the genetic makeup of the host is a major factor that impacts the course of the HCV infection. The hepatitis A virus cellular receptor 1 (HAVCR1) gene, a member of the T cell immunoglobulin and mucin (TIM) gene family, shows a variable susceptibility towards the different genotypes of HCV [25]. The IL28B genotype CC was found to be linked with more infections from genotype 3 than genotype 1 or 4 in HIV-coinfected patients [26]. The result of a study of HCV-genotype-1-infected individuals showed that polymorphisms of the IL28B (IFN-λ3) gene is protective against chronic Hepatitis C and a predictor of response to interferon-based therapy [27]. The genetic variation in the IFN-λ3 gene could be associated with a spontaneous clearance of an acute HCV infection. Genomic wide studies have shown that rs12979860 [28] and rs8099917 [29] are associated with a spontaneous resolution of an HCV infection. Furthermore, IFN-λ polymorphism is linked to a persistence of HCV; there is an upregulation of USP18, an inhibitor of the interferon-stimulated gene (ISG) [30,31]. Wojcik et al. demonstrated the association of single-nucleotide polymorphism rs6880859 and rs953569 in the HAVCR1 gene correlated with an increased persistence of HCV in an individual of African and European descents respectively [25]. Stimulation of Drp1 by HCV is a major factor in HCV virulence, which leads to an uneven fragmentation of mitochondria [23].

## 3. Clinical Manifestations

The hepatitis C virus is associated with two forms of disease progression: acute and chronic hepatitis C viral infections. Although the majority of individuals with an acute HCV infection are asymptomatic, up to 30% of individuals with an acute HCV infection are symptomatic [32]. These symptoms may include weakness, anorexia, right upper quadrant abdominal pain, dark-colored urine, spider angioma, edema of lower extremities, and jaundice with serum levels of alanine aminotransferase (ALT) and aspartate aminotransferase (AST) above 10 times the normal range. The consequence is damage to the liver tissue [33]. However, 30% of individuals with an acute HCV infection will undergo a spontaneous clearance of the infection within 6 months of infection [34]. In self-containing acute infections, the levels of both ALT and HCV RNA drop with time [9]. Anti-HCV antibodies are detectable within 8–12 weeks of HCV exposure with serum HCV RNA levels fluctuating during the acute phase of HCV infection [34]. Late or undetectable levels of anti-HCV antibodies could lead to major liver damage. More than 70% of individuals with an acute HCV infection develop a chronic HCV infection characterized by the presence of HCV RNA in the blood for more than 6 months after the onset of an HCV infection. The risk factors for developing a chronic HCV infection include age at the time of infection, male gender, immunocompromised state, a coinfection with HIV, having asymptomatic acute HCV infections, and ethnicity of the infected individual (Reviewed in Reference [9]). Extrahepatic manifestations of a chronic HCV infection include mixed cryoglobulinemia, membranoproliferative glomerulonephritis, lichen planus, vitiligo, keratoconjunctivitis sicca, and lymphoma [34,35]. Levels of ALT also plays a role in the rate of disease progression. If serum ALT is at normal levels, then the disease progression is gradual compared with the significantly raised serum levels of ALT [36]. Approximately one in three individuals with a chronic HCV infection are most likely to develop liver cirrhosis following years of progressive fibrosis of the liver. The development of cirrhosis increases the risk of developing hepatocellular carcinoma [34], which is also associated with a chronic HCV infection under various conditions. An HCV coinfection with HBV leads to a higher chance of developing HCC [36]. Also, coinfection with HIV and host genetic factors are associated with HCV-associated HCC development.

## 4. HCV and Host Interaction

**Figure 1:**
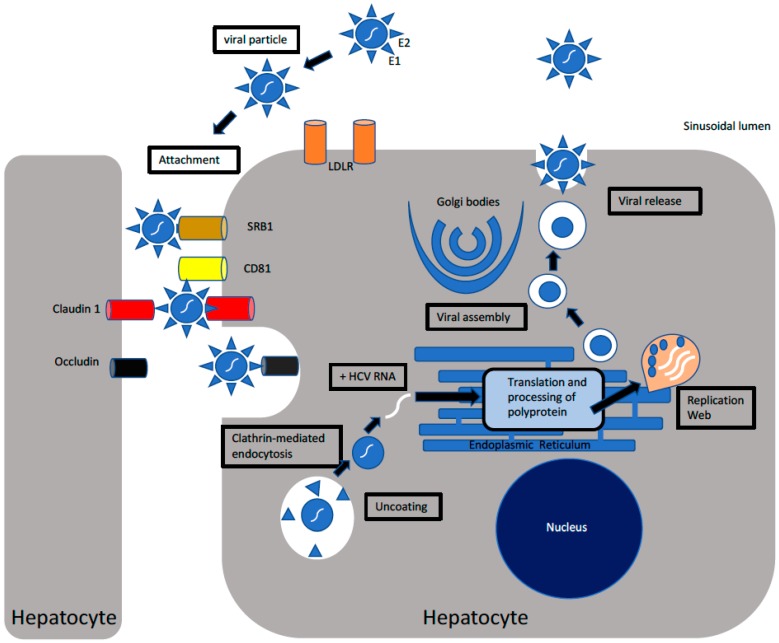
The replication of hepatitis C (HCV): The virus via its envelope glycoproteins attach to host cellular receptors such as claudin-1, epidermal growth factor receptor (EGFR), scavenger receptor class B type 1 (SRB1), cluster of differentiation (CD81), low density lipoprotein receptor (LDLR), and DC-SIGN (Dendritic Cell-Specific Intercellular adhesion molecule-3-Grabbing Non-integrin) to attach and subsequently gain entry into host cells. Following attachment, HCV entry occurs via clathrin-mediated endocytosis, wherein HCV undergoes uncoating to release the nucleocapsid into the cytoplasm. HCV RNA is released into the cytoplasm, where it is exposed to host immune machinery. HCV RNA translation via an Internal Ribosome Binding Site (IRES) at the rough endoplasmic reticulum (ER) gives rise to a large polyprotein that undergoes processing into nonstructural and structural proteins. Nonstructural protein NS4B induces the formation of a membranous replication web, where viral RNA replication occurs via the action of RNA-dependent RNA polymerase. The nascent positive sense RNA genome is used for the production of viral proteins, further RNA replication, or the formation of new virions. Utilization of fatty acid pathways along with structural proteins culminate in viral assembly and release.

HCV is a lipid centric virus with two envelope glycoproteins E1 and E2, which mediates viral entry into permissible host cells. These two glycoproteins interact with CD81 and various other surface proteins such as claudin-1 [1,37,38], occludin, and epidermal growth factor receptor to access the cell [39]. Clathrin-mediated endocytosis is required for viral entry into the target cell, wherein the nucleocapsid is released into the cytoplasm. Following the release of nucleocapsid in the cytoplasm, the genomic material of HCV is exposed to the host’s immune machinery. Positive-strand RNA-bearing Internal Ribosome Binding Site (IRES) is used for the translation of HCV proteins. HCV translates a large polyprotein which is broken down by cellular (Signalase and Signal peptide peptidase) and viral (NS2 and NS3/4A) proteases into structural and nonstructural proteins during ER-related processing [40]. The NS5B and helicase domain of NS3 are regulators of HCV replication, which helps to unwind and stabilize the HCV RNA in a replication complex [41,42]. NS4B helps in the formation of the compartments for HCV replication by producing “membranous web” structures [14]. Certain host factors also assist in HCV replication such as microRNA-122 which binds to IRES to increase the efficiency of translation and Cyclophilin A, which interacts with NS5A and NS5B to increase HCV replication [14]. HCV also uses fatty acid pathways and very low density lipoprotein (VLDL) production for assembly and release [43]. Figure 1 illustrates the life cycle of HCV, highlighting the major steps I HCV replication including HCV attachment and entry into the host cell, the translation of HCV RNA to yield a large polyprotein that is processed into ten HCV proteins, HCV RNA replication, and viral assembly and release.

### 4.1. Innate Immune Response in HCV Infection

**Figure 2:**
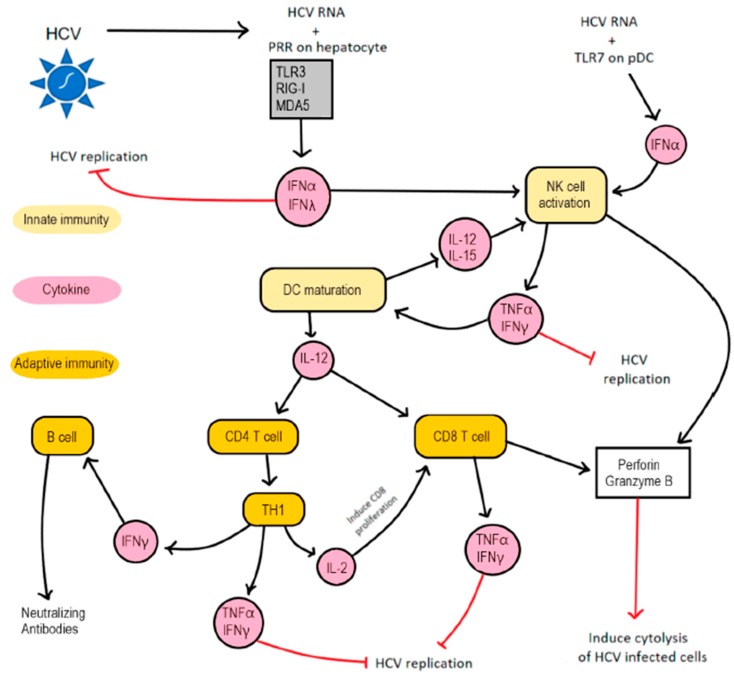
A host immune response to an HCV infection: The interaction between HCV and hepatocytes induces innate and adaptive immune responses. During an HCV infection of hepatocytes, HCV RNA engages TLR3, RIG-I, and MDA5 on infected hepatocytes as well as TLR7 on pDC to induce the secretion of type I and III interferons. Type I and III IFN inhibit HCV replication and activate NK cells. Activated NK cells produce IFN-γ and TNFα, which induce DC maturation and inhibit HCV replication. Matured DC produce IL-12 that induce the differentiation of CD4 T cells and CD8 T cells into Th1 cells and Cytotoxic T cells, respectively. Additionally, IL-12 and IL-15 secreted by DC activate NK cells. Th1 cells secrete IL-2, IFN-γ, and TNFα. IL-2 induce the proliferation of CD8 T cells, whereas IFN-γ and TNFα inhibit HCV replication without inducing a cytolysis of HCV-infected cells. Furthermore, IFN-γ produced by Th1 cell induce the differentiation of B cells into plasma cells that produce neutralizing antibodies. Finally, perforin and granzyme B produced by CTL and activated NK cells induce the cytolysis of HCV-infected cells.

During an acute infection with HCV, viral RNA is detected in the blood within 1–2 weeks postinfection [44] and activates the innate and adaptive arms of the immune response. Figure 2 describes the innate and adaptive immune responses against HCV. The innate immune response includes type I interferon in infected cells [45], which induces double-stranded RNA-dependent protein kinase (PKR) and other genes to induce apoptosis of infected hepatocytes, as well as to inhibit viral replication [46]. Compared to HBV, HCV initiates a better innate response due to the exposure of its genetic material in the cytoplasm. The major players in HCV-induced immune responses are interferons (IFNs) I and III, interferon stimulated genes (ISGs), NK cells, T cells, and antibody-type responses. Following an uncoating of HCV, TLR3 and retinoic acid-inducible gene-I (RIG-I)-like receptor (RLR) on HCV-infected hepatocytes sense HCV and respond by generating type I and III IFN that inhibit the replication of HCV as well as activate NK cells. An interaction between the HCV dsRNA replication intermediate and ssRNA with RIG-I and melanoma differentiation-associated gene 5 (MDA-5) activate the Toll/IL-1R- (TIR) containing adapter inducing IFN-β (TRIF) and mitochondria antiviral signaling protein (MAVS), which phosphorylate IFN regulatory factor 3 (IRF3) and IRF7 to induce type I and III IFN production [47,48]. Additionally, a TLR3-mediated innate immunity is induced when TLR3 interacts with the dsRNA replication intermediate to activate TIR that phosphorylates IRF3 [31]. Type I (IFN-α and IFN-β) and type III (IFN-λ) interferon via their respective receptors phosphorylate STAT-1 and STAT-2 to generate IFN-stimulated gene factor 3 (ISGF3), a transcription factor that translocate into the nucleus, where they play a role in producing IFN-stimulated antiviral genes [31,49]. It is important to note that IFNLR, a receptor for type III IFN, is expressed on epithelial cells, hepatocytes, and DC. Thus, a defect in type I and III IFN signaling renders hepatocytes highly susceptible to HCV [31,50]. It is important to note that, during HCV infection, the levels of IFNs and ISGs are always upregulated in the cell. Generally, they have an inflammatory response towards the threat, but in the case of HCV, components like ubiquitin-specific peptidase 18 (USP18) and ISG15 negatively regulates the downstream signaling pathways of interferon signaling and helps in the longer persistence of HCV in the cell [30]. USP18 downregulates the production of IFN-α through an interaction with IFNAR signaling [51]. ISG15 is abundant in the cell during an HCV infection, and it also stabilizes USP18 which relates to poor IFN-α processing [52].

The cellular innate immune response against HCV is mediated by NK cells, which are paramount in an HCV infection. NK cells constitute about 30–50% of intrahepatic lymphocytes [53]. It is important to note the different subset of NK cells on the basis of the expression of CD16 and CD56. CD16+CD56^dim^ NK cells are more cytolytic in nature, whereas CD16-CD56^bright^ NK cells usually have a predominantly noncytolytic phenotype [31]. NK cells secrete TNF-α and IFN-γ that inhibit HCV replication as well as cytolytic enzymes that destroy HCV-infected host cells. The cytolytic action of NK cell-released perforin/granzyme could cause collateral damage to host tissues. An upregulation of KIR receptors which are found on NK cells and are markers for lysis of the target cells is seen during an HCV infection, indicating the importance of NK cells [54]. Thus, NK cells through the cytolysis of infected cells, cytokine production, and the activation of T cells [55,56,57] results in an initial reduction in the systemic HCV viral load. This is followed by the activation of adaptive immunity, during which virus-specific CD4^+^ T, CD8^+^ T, and B cells are induced by antigen presenting cells (APCs), specifically DCs. DCs bind to the Nkp30 receptor on NK cells and produce IL-12 and IL-15 that activates an NK cell, and activated NK cells secrete IFN-γ and TNFα that reciprocally enhance the maturation and antigen presentation of DC [58]. Natural killer T (NKT) cells are another group of innate cells, which comprise 26% of intrahepatic lymphocytes [59,60] and secrete IFN-γ, TNFα, and IL-2 [60]. Though its precise role in a chronic infection is yet unclear, there are indications that NKT cells may influence the balance of T_H_1 versus T_H_2 responses to an HCV infection [61]. While one report indicates an increase in NKT cell frequency in the liver of patients with a chronic HCV infection [62], another has observed a decrease [63]. Irrespective of the numbers, NKT cells from HCV patients show an altered production of IL-13 [64]. IL-13 is a Th2 cytokine that shows some functional redundancy with IL-4 and has also been implicated in regulating cell-mediated immunity and allergic asthma [65].

CD11c^+^ myeloid DC (mDC1), CD141^+^ myeloid DC (mDC2), and plasmacytoid DC (pDC) are DC subsets involved in producing cytokines in response to an HCV infection. IL-12, IFN-λ, and IFN-α are produced by mDC1, mDC2, and pDC respectively in response to an interaction between HCV pattern-associated molecular patterns (PAMP) and pattern recognition receptors on DC. These cytokines possess immunostimulatory properties [31]. mDC presents viral antigenic peptides to naïve T helper cells in the lymph nodes, thus skewing them to differentiate into T_H_1 cells [66]. In addition to the stimulation provided by an HCV antigen presentation on DCs, these activated T_H_1 cells secrete IFN-γ and IL-2, which enhance the antigen presenting capabilities of DC and the proliferation of HCV-specific CD8+ T cells respectively [45,67].

### 4.2. Adaptive Immune Response in HCV Infection

Neutralizing antibodies to HCV appear within 8-12 weeks and interfere with the interaction of CD81, LDLR, SRB1, and claudin-1 with HCV envelope glycoprotein E1 and E2 in early acute HCV infection. As such, neutralizing antibodies inhibit the binding of viral envelopes to host cellular receptors [31,68]. Additionally, neutralizing antibodies to HCV inhibits the viral and cellular factors that promote HCV entry into host cells [69]. HCV E1 and E2 are the targets of neutralizing antibodies; however, antibodies are short-lived and are not persistent during the chronic stages of the infection [70]. A replication-competent HCV cell culture (HCVcc) and HCV pseudopeptide (HCVpp) are in vitro neutralization assays for evaluating the antibody neutralization of HCV [69]. Using a multiplexed flow-cytometric microassay to measure anti-HCV IgG response to HCV core and nonstructural HCV recombinant proteins (NS3, NS4, and NS5), Araujo et al. demonstrated that a chronic HCV infection was associated with higher levels of anti-HCV IgG response than in an acute HCV infection [71]. Moreover, Filomena et al. demonstrated that a multiplex HCV serological assay could discriminate between acutely and chronically HCV-infected patients [72]. A mutation affecting the binding site of E2 on CD81 could result in the development of resistance to broad neutralizing antibodies in an HCV infection [68,73]. Because of the hypervariable regions in E1 and E2 glycoproteins and high mutation rates, T cell and B cell responses are short and quite inefficient. Due to a direct cell to cell transmission of HCV, it often escapes the antibodies and is difficult to neutralize [70]. Neutralizing antibodies are thought to have a lesser role in controlling an HCV infection as they were detected more in chronic stages rather than after acute infections [74].

CD4^+^ T cells provide help for priming CD8^+^ T cell response and activating DC via the action of IL-2 and IFN-γ. The presence of HCV-specific CD4^+^ T cell responses during the acute phase of an HCV infection is associated with the control of viral replication. If the CD4^+^ T cell response is sustained and maintained, there is a permanent clearance of HCV; however, if there is a loss of CD4^+^ T cell responses, a rebound viral replication or viremia, occurs leading to viral persistence [75,76]. In chronic HCV infection, CD4^+^ T cells have a limited functionality due to an impaired proliferative capacity as a consequence of the HCV core-mediated suppression of IL-2 secretion [77]. Likewise, an interaction between an HCV core and DC results in a skew in the T cell response to IL-4 and IL-10 producing T cell due to the HCV core-mediated inhibition of IL-12 production [78]. Although the expression of coinhibitory molecules on activated T cells is protective, an overexpression of coinhibitory receptors in the setting of a low expression of CD127 on HCV-specific CD4^+^ T cells is associated with a persistent HCV infection, in which, the loss of CD4^+^ T cell negatively impact on the functionality of CD8^+^ T cells [79].

In an acute HCV infection, HCV-specific CD8^+^ T cells perform cytolytic and noncytolytic functions to mediate viral clearance. The CD8^+^ T response is enhanced via the assistance of CD4^+^ T cells during the acute stages of infection. The HCV-specific CD8^+^ T cells leave the lymph nodes and traffic to the liver where they mediate the clearance of HCV-infected hepatocytes by recognition of HCV-antigenic peptides loaded on human leukocyte antigen (HLA) class I on their surfaces [45]. The cytolysis of HCV-infected hepatocytes is mediated by perforin and granzyme B secreted by CTL. Noncytolytic HCV clearance is mediated by IFN-γ and TNFα that favor the generation of antiviral microenvironment [76], in which viral replication is inhibited without killing the infected cell. HCV-specific T cell responses and the secretion of IFN-γ have been found to correlate with a decrease in the HCV RNA load [44,80]. A sustained vigorous HCV-specific CTL response is associated with the resolution of an acute HCV infection; however, suboptimal performing CTL correlates with viral persistence [81,82]. While CD8^+^ T cells are the primary effector cells, in the absence of a strong HCV-specific CD4^+^ T cell response, their ability to keep up with viral replication is lost and a persistent infection develops [83]. HCV-specific CD8^+^ T cells exposed to high viral loads in a chronic HCV infection exhibit a reduced ability to both proliferate and produce IFN-γ [76]. Exhausted HCV-specific CD8^+^ T cell expresses PD-1, 2B4, TIM-3, CTLA4, or CD160 with a reduced expression of CD127 [79].

HCV-infected individuals who cleared the infection in the acute phase demonstrated the presence of significant levels of HCV-specific CD4^+^ and CD8^+^ T cells. It has been shown that HCV infection does not result in the development of sterilizing immunity but rather the memory CD4^+^ and CD8^+^ T cells provide protective immunity with CD4^+^ T cells providing help to CD8^+^ T cell to respond to viral escape mutants in class I MHC-restricted epitopes [84,85]. T cells are involved in the immunopathogenesis of an HCV infection of the liver. The cytolytic mechanism of viral clearance involves the activity of Fas ligand, perforin, granzyme, and TNF-related apoptosis inducing ligand (TRAIL). A Fas-FasL system in an HCV-infected liver is mediated by HCV-specific CD8^+^ T cells that express FasL HCV-infected hepatocytes that upregulate the expression of FasL, which interact with Fas receptors to induce apoptosis of HCV-infected hepatocytes. The Fas-mediated apoptosis involves the activation of caspase-8 and caspase-9 and the subsequent activation of downstream caspase-3, -6, and -7 that cause cell death [86]. Perforin and granzyme B released by activated CTL induced the apoptosis of HCV-infected hepatocytes via granzyme B cleaving pro-caspase [87,88]. Liver damage occurred when CTL induced hepatocyte apoptosis with the subsequent development of liver fibrosis and HCC. Some of the hepatocytes may be damaged via CTL-mediated by standing killing [88]. Thus, CD8^+^ T-cell-induced Fas/FasL pathways induce immunopathogenesis in HCV-infected livers by killing infected and noninfected cells.

Many studies have shown that HLA class II alleles are associated with spontaneous viral clearance and the persistence of HCV. HLA-DRB1*0101 [89,90], HLA DRB1 *0501 [91], and HLA DQB1*0301 [89,92,93] are associated with the spontaneous clearance of HCV. However, HLA DQB1*0201 [91] and HLA DRB1*0301 [94] are associated with a persistence of HCV. Similar to studies of association between HLA class II alleles and HCV outcomes, some HLA Class I alleles have an effect on the outcome of an HCV infection. HLA-A03 [95], HLA A11 [96], HLA B27 [95], and HLA B57 [96,97] are associated with the spontaneous clearance of HCV. However, HLA B08 [95] and HLA Cw*04 [96] are associated with a persistence of HCV.

### 4.3. Effect of HCV on Myeloid Cells

Myeloid cells and their phagocytic activities were first discovered by Élie Metchnikoff in 1884 [98]. Myeloid cells comprise various cellular subtypes and are operationally divided into mononuclear and polymorphonuclear cells. Mononuclear phagocytes include macrophages, which are found in essentially all tissues. Inside the tissues, macrophages perform several important functions such as a regulation of tissue homeostasis, immune surveillance, and inflammation [99,100]. Mononuclear phagocytes also include dendritic cells (DCs), which consist of distinct subsets. Classic DCs (cDCs) form the predominant DC subset and their main function is to sample antigens in tissues and migrate to local draining lymph nodes to induce antigen-specific T cell immunity or tolerance [101,102]. Plasmacytoid DCs (pDCs) are another DC subset that produce high levels of interferon-α (IFN-α) after sensing an infection [103]. There is another subset of myeloid cells, known as Myeloid-derived suppressor cells (MDSCs), which rapidly expand during chronic infection, cancer, obesity, and trauma. MDSCs are a heterogeneous population of cells that are defined by their myeloid origin, immature state, and ability to potently suppress T cell responses [104]. MDSCs broadly include immature myeloid progenitors, monocytic-MDSCs, and granulocytic MDSCs. MDSCs are viewed as distinct from terminally differentiated myeloid cells such as macrophages and DCs; however, it is important to recognize that macrophages can also exhibit T-cell-suppressive activity [105,106]. MDSCs suppress the effector function of target cells through multiple mechanisms (reviewed in detail in References [107,108]). These mechanisms include the production of Nitric Oxide (NO) and reactive oxygen species (ROS) which prevent T cell activation in response to cognate antigen and induce T cell apoptosis. MDSCs also secrete Arginase-1 which depletes L-arginine required by T cells to proliferate. MDSCs can also produce immunosuppressive cytokines, such as IL-10 and TGF-β. It has been reported that HCV infection generates CD33+CD14+CD11b+HLADR-/low M-MDSCs, which effectively suppress T cell responses through the production of ROS [109]. Another study has reported an expansion of M-MDSCs (CD14+CD33+CD11b+HLA-DR-/low) in the peripheral blood of chronic HCV patients [110]. These HCV-induced M-MDSCs have high levels of pSTAT3 which drives the expression of IL-10. Importantly, coculturing MDSCs derived from HCV-infected patients or treated with HCV core protein with healthy peripheral blood mononuclear cells (PBMCs) resulted in a significant increase in the numbers of CD4+CD25+Foxp3+ regulatory T cells. In addition, a depletion of MDSCs from PBMCs of HCV patients significantly increased the interferon-γ production by CD4^+^ T effector cells. In an important study, Zeng et al. showed that MDSCs frequency was significantly higher in treatment-naive chronic HCV patients compared to healthy subjects and chronic HCV patients that responded to PEG-Interferon-α/Ribavirin therapy [111]. The MDSCs frequency in treatment-naive chronic HCV patients positively correlated with HCV RNA. An increased frequency of MDSCs in treatment-naive chronic HCV patients was significantly associated with decreased T cell receptor (TCR) ζ expression on CD8^+^ T cells. TCR ζ expression was restored by L-arginine treatment in vitro. The mechanisms by which HCV induces MDSCs are poorly understood. Wang et al. have shown that HCV-infected cells can secrete HCV RNA-containing exosomes. These exosomes after being taken up by monocytes to promote the expansion of M-MDSCs. Importantly, this M-MDSC expansion is mediated by a downregulation of the miR-124 expression [112].

Peripheral blood DC include myeloid DC and plasmacytoid DC, and peripheral blood dendritic cells (PBDCs) are susceptible to an HCV infection [113]. HCV is known to target DC functions to suppress the generation of strong antiviral innate and adaptive immune responses. Although DCs can be infected by HCV at very low levels, it is less likely that the virus utilized DCs to produce viral progeny [113,114,115]. An infection and replication of HCV in PBDC dysregulates the allostimulatory function and IFN-α production by mDC and pDC respectively in an HCV chronic infection [113]. However, there are some observations that might support the role of DCs in the dissemination of an HCV infection. The HCV envelope glycoprotein E2 as well as HCV virions isolated from HCV-infected patients have been shown to bind specifically to DC-SIGN, a C-type Lectin receptor present on the surface of DCs. Thus, it may be possible that blood DCs or hepatic DCs in the liver sinusoids bind to circulating HCV and transmit the virus to hepatocytes. Consistent with this, the HCV pseudo virus was shown to bind DC-SIGN expressed on monocyte-derived DCs and was transmitted efficiently when cocultured with the human hepatocellular carcinoma cell line Huh7, a cell line that supports HCV pseudovirus entry and productive infection [116,117]. In terms of HCV affecting DC frequencies, multiple studies have reported lower numbers of blood mDCs and pDCs in HCV-infected patients compared to healthy controls [118,119,120]. In an HCV infection, blood DC subsets are enriched in the liver [121], which explains why their numbers are reduced in the blood. However, lower numbers of circulating DCs have also been observed in non-HCV related liver diseases such as granulomatous hepatitis or primary biliary cirrhosis, suggesting that the low DC count in virus-related liver diseases might be a common, nonspecific feature of inflammation. Interestingly, DCs exposed to the serum of HCV-infected patients in vitro show a reduced ability to migrate in response to CCL21, a chemokine that recruits DCs to draining lymph nodes via CCR2-CCL21 axis [121]. This suggests that hepatic DCs could be trapped in the liver and unable to migrate to draining lymph node and prime antiviral T cell responses; however, it needs to be confirmed.

### 4.4. Effect of HCV on Lymphoid Cells

It has been demonstrated that HCV can infect lymphoid cells via its interaction with CD81. Lymphotropic HCV strains can infect and replicate in B cells and T cells [122]. These strains could be released by HCV-infected PMBC with a role to play in HCV persistence. HCV infection and replication in CD4^+^ T cells result in a reduced proliferative capacity, an enhanced Fas-mediated apoptosis, and the suppression of IFN secretion [87,123], whereas the infection of B cells via the interaction with CD21, CD19, and CD81 complexes [124] can result in malignant lymphoma, since peripheral B cells serve as a reservoir for HCV [125]. Furthermore, it has been reported that the coexpression of CD5 and CD81 enhances the tropism of HCV for T cells [126]. The replication of HCV in T cells is associated with a reduction in IFN-γ production due to the inhibition of STAT1 activation as well as an enhanced susceptibility to Fas signaling [127].

### 4.5. Effect of HCV on Nonimmune Cells

Nonimmune cells affected by an HCV infection include hepatic stellate cells (HSC), hepatocytes, and liver sinusoidal endothelial cells (LSEC). HCV-infected hepatocytes secrete type I and III IFN that trigger DC, HSC, and Kupffer cells to produce IL-12, IL-15, and IL-18 to recruit IFN-γ-producing NK cells, whereas type I and III IFN induce LSEC to secrete CXCL10 that recruit activated T cells to the liver [31,60]. HSC and LSEC are nonimmune cells resident in the liver that exhibit antiviral effects in response to an HCV infection. HCV RNA induces a TLR3-mediated secretion of IFN-λ when it engages TLR3 expressed on HSC [128], whereas an interaction between HCV RNA and TLR7 expressed on LSEC generates type I and III IFN [129,130]. It is important to note that HSC and LSEC do not support the efficient replication of HCV [31]. Resident cells in the liver such as LSEC, Kupffer cells, and hepatic stellate cells promote a tolerogenic microenvironment in the liver by inducing tolerance to infiltrating effector CD4 T cells and CD8 T cells [86]. The expression of TGFβ by hepatic stellate cells may favor the generation of Th2 immune response with production of IL-10 as well as render other liver APCs tolerogenic [131].

## 5. Mechanisms Responsible for the Development of Chronic HCV Infection

During chronic infections, an important feature is that immune responses towards targeted viruses are impaired or altered. Several mechanisms have been proposed for the failure in host immune responses to clear HCV infection. (1) The escape due to genetic variations, (2) the suppression of immune responses by HCV proteins, (3) the inhibition of innate immune responses during a chronic HCV infection, (4) the dysfunction of T lymphocytes, and (5) the involvement of Regulatory T cells (T_regs_) in chronic HCV infection are factors that contribute to an impaired or altered immune response against HCV. An immunological escape due to genetic variations is a major immune evasion strategy used by HCV. Furthermore, the rapid diversification of the HCV genome attributed to a high replication rate and an intrinsic lack of proofreading by HCV RNA-dependent RNA polymerase contributes to an evasion of immunosurveillance and the emergence of quasispecies [132,133,134]. In each HCV-infected individual, various closely quasispecies-related but nonidentical viral genomes, are subjected to continuous mutation, competition, and selection [45]. Likewise, the hypervariable region 1 (HVR 1), a small fragment spanning 27 amino acids of E2 on a highly variable region of HCV genome, is a sequence mutation that plays a role in evading neutralization by HCV-specific antibodies [45,135,136]. HCV mutations located in NS3 and NS5 are targeted by CD4^+^ T cells, and these escape mutants to HCV-specific CD4^+^ T cell responses contribute to immune evasion [137]. Additionally, HCV genomic mutations in regions of the CD8^+^ T cell epitope have also been known to affect virus-specific CD8^+^ T cells by decreasing the T cell receptor (TCR) recognition of mutated peptides, impairing the binding affinity between epitope and MHC molecule and weakening the ability of proteasomes to process HCV antigens [138,139,140]. An analysis of the sequencing spanning parts of nonstructural protein in a chronic HCV patient revealed sequence polymorphisms in CD8 restricted epitopes [141,142].

HCV proteins play a significant role in chronic HCV infection. They exhibit an immunosuppressive activity on DC, NK cells, and T cells, which contributes to the establishment of a chronic HCV infection. HCV proteins may interfere with endogenous IFN and toll-like receptor (TLR) responses. NS3/4A serine protease has been shown to interfere with RIG-I and TLR3 signaling, consequently interfering with endogenous IFN production [143,144,145]. HCV core protein degrades STAT1, and as such, inhibits the activation of STAT1 [146,147]. It also inhibits interferon-stimulated gene factor 3 (ISGF3) via the initiation of suppressors of cytokine signaling 3 (SOCS-3) expression, which impedes the binding of ISGF3 to the IFN-stimulated response elements (IRES) in the promoter regions of the ISG [148,149]. The HCV NS5 protein impairs the ability of pDCs to produce IFN-α [118,150,151]. HCV core and E1 proteins inhibit DC maturation, which in turn, impairs the ability of DC to activate T cells [152]. Furthermore, HCV core protein interacts with globular domain of C1q receptor (gC1qR), a complement receptor for C1q on DCs, to suppress production of IL-12, a key cytokine required for Th1 differentiation [153]. Likewise, the HCV core protein interacts with gC1qR on monocyte-derived DC to reduce IL-2 expression, consequentially inhibiting T cell proliferation [154]. Furthermore, the HCV core-mediated suppression of IL-2 production could contribute to an impaired differentiation of the central memory HCV-specific CD8 T cells into effector HCV-specific CD8^+^ T cells [86,155]. The HCV core also downregulates MHC and costimulatory molecule expression on DC, resulting in an impaired ability to prime HCV-specific CD4^+^ and CD8^+^ T cell response and facilitating the induction of IL-10 producing T cells [156]. Moreover, the interaction of HCV core with gC1qR on macrophages induces the expression of A20, a negative regulator in macrophages with a consequential reduction in the secretion of IL-1 and IL-6 [157]. HCV core protein interaction with gC1qR on monocyte-derived DC results in an inhibition of TLR-mediated IL-12 production and a reduced IFN-γ production by allogeneic CD4^+^ T cell with a consequential impairment of Th1 differentiation of CD4^+^ T cells [153]. The binding of HCV E2 proteins to CD81 on NK cells was shown to be associated with an impaired NK cell-mediated cytolytic function and an impaired IFN-γ production [158]. However, Yoon et al. contradicted this concept of an impairment of the NK cell function via HCV E2-associated crosslinking of CD81, as they demonstrated that HCV E2 from infectious virions was inefficient in inducing a CD81 crosslinking on NK cells [159]. HCV core 35–44 is a HLA-A2-restricted T cell epitope that increases the stability of HLA-E, a known ligand for the inhibitory receptor CD94/NKG2A on NK cells, which results in a blockade of NK-cell-mediated cytolysis [160]. The HCV core protein also increases an expression of MHC class I on infected cells via the enhancement of TAP1 expression, which results in a resistance to the NK cell killing of infected cells [161]. The HCV NS4A/B protein can block HLA class I expressions on surfaces of infected cells by inhibiting the endoplasmic reticulum–Golgi traffic [162], which results in an impaired recognition of HCV-infected hepatocytes by HCV-specific CD8^+^ T cells. The lack of key activation signals and cytokines from dysregulated APCs may play a role in generating functionally impaired T lymphocytes during a chronic HCV infection. HCV proteins may influence the regulation of apoptosis of infected cells, thereby helping it escape host responses. HCV core, NS3, NS5A, and NS5B may trigger the apoptosis of mature DCs [163]. Some reports show the HCV core protein inducing Fas-mediated apoptosis [164,165], and others reports emphasize their role in inhibiting TNF-α-mediated apoptosis via its upregulation of cellular FLICE (FADD-like interleukin-1β converting enzyme) like inhibitory protein (cFLIP) [166]. Additionally, HCV E2 can also prevent apoptosis through the inhibition of TRAIL-mediated cytochrome c release from mitochondria [167].

The inhibition of innate immune cells during an HCV infection is an immune evasive strategy employed by HCV. The immune cells of the liver comprise significantly of Kupffer cells (macrophages), NK, NKT, and different DC subsets. It has been demonstrated that HCV can interfere with the activation and function of immune cells that mediate innate immunity. The presence of adequate NK cell response and matured DCs are critical for inducing an effective HCV-specific adaptive response [56], as a reciprocating activating crosstalk between DCs and NK cells is vital in driving the antiviral innate immune response [168]. IFN-α stimulated DCs are induced to express MHC-class-I-related chain A/B that ligates with NKG2D-activating receptors on NK cells to induce NK cell activation. Additionally, DCs secrete IL-12, IL-15, and IL-18 that activate NK cells [169]. A reduction in NK cell frequency and cytotoxic function has been reported in the peripheral blood and livers of HCV-infected individuals. An increased expression of inhibitory receptors (CD94/NKG2A) on NK cell in chronic hepatitis C patients [170] coupled with a reduction in the proportion of natural cytotoxicity receptors (NKp30 and NKp46) on NK cells has been noted in HCV patients [171]. NK cell cytotoxicity capabilities are impaired, and they secrete a significant amount of IL-10 and TGFβ that skew the adaptive immune response by downregulating the function of DC [170,172]. It has been demonstrated that IFN-α therapy in patients with chronic HCV infection can restore NK cell functionality, thus indicating that HCV may suppress NK cell functions during the chronic infectious phase [173]. It has been shown that coculturing CD33+ PBMCs with HCV-infected Huh7.5.1 cells expanded CD33+CD11b^low^HLA-DR^low^ MDSCs. NK cells cocultured with these MDSCs produced lower amounts of IFN-γ, with no effect on granzyme B production or cell viability. Importantly, this suppression of NK cell–derived IFN-γ production was mediated by CD33+CD11b^low^HLA-DR^low^ MDSCs via an arginase-1–dependent inhibition of the mammalian target of rapamycin activation which results in a reduced protein translation [174].

DCs play an important role in initiating adaptive immune response to virus; however, a reduced frequency of mDCs and pDCs is observed during an HCV infection [118,120], and this can be correlated with an impaired capacity of these cells to activate T cells [118]. It has been demonstrated that pDC with a reduced capability to produce IFN-α has been observed from the blood of HCV-infected individuals [175]. Furthermore, studies have shown that mDCs in the setting of an HCV infection have reduced the expression of costimulatory molecules (CD83 and CD86) and impaired the ability to secrete IL-12, which results in an impaired ability of mDC to present antigen to T cells [176,177]. Likewise, an HCV-associated DC dysfunction could be attributed to the induction of pDC apoptosis, impaired DC trafficking due to a nonresponse to CCL21, and a reduced activation of DC via the downregulation of pattern recognition receptors [31,177,178]. Dysfunctional DC in HCV infection are unable to induce cytokine-dependent NK cell maturation and T cell priming [156,179]. Additionally, HCV-infected DC induces immune tolerance, since they secrete significant amounts of IL-10 [118,180] with a consequential suppression of T cell responses. This is likely to result in a failure to maintain a sustained HCV-specific T cell immune response during chronic infection.

The dysfunction of T cells occurs during a chronic HCV infection. There is evidence that suggests that T cells, especially CD8^+^ T cells need to be fully functional in order to successfully control chronic viral infections. In recent years, a greater emphasis has been put on the need for a polyfunctional population of T cells, which can be correlated to a better control of viral load [181]. Due to the inability of the immune system to control the viral load during chronic infections, significant levels of viral loads correlate with a persistent exposure of T cells to HCV, which render T cells exhausted [182]. Data that has been derived from the study of the lymphocytic choriomeningitis virus (LCMV) murine model indicates that an impairment of CD4^+^ and CD8^+^ T cells function occurs. These exhausted T cells have a reduced capability to produce proinflammatory cytokines, an impaired cytolytic function, and a reduced capacity to proliferate [183,184,185]. Reports show HCV-specific CD8^+^ T cells in chronic infections to be functionally impaired with respect to IFN-γ production and proliferation as well as exhibit a reduction in both cytotoxicity and degranulation potential [186,187,188]. Impaired HCV-specific CD8^+^ T cells were also observed to undergo massive apoptosis in the liver during the chronic phase [189]. Thus, the impaired function of both CD4^+^ T cells and CD8^+^ T cells correlate with the persistence of HCV infection [75]. The phenotype of exhausted T cells, particularly CD8^+^ T cells, in a chronic HCV infection overexpress coinhibitory receptor molecules, such as PD-1, CTLA-4, LAG-3, 2B4, CD160, and TIM-3 in viremia [185,190,191,192]. The co-expression of multiple distinct inhibitory receptors was found to determine the level of CD8^+^ T cell exhaustion as well as the severity of infection [192]. PD-1 interacts with its ligands PD-L1 (or B7-H1) and PD-L2 (B7-DC) to regulate immune responses in both lymphoid and nonlymphoid organs [193]. It is of note that the sustained expression of PD-1 on HCV-specific CD8^+^ T cells has also been observed during a chronic infection with HCV [194,195,196]. Additionally, high PD-1 levels on HCV specific T cells during an acute infection may determine the viral persistence and progression to a chronic infection [194,197]. These findings, therefore, implicate the PD-1 pathway as a major determinant controlling T cell exhaustion during a chronic viral infection. Another phenotypic feature of exhausted CD8^+^ T cells when compared with memory CD8^+^ T cells is the lowered expression of IL-7Rα (CD127) and IL-2Rβ (CD122), the receptors for the homeostatic cytokines IL-7 and IL-15, respectively [198,199]. These virus-specific CD8^+^ T cells from a chronic infection also lacked responsiveness to IL-7 and IL-15 in vitro and did not undergo homeostatic proliferation. Similarly, intrahepatic HCV-specific CD8^+^ T cells were found to express significantly reduced levels of CD127 [196]. These results suggest that the development of an effective memory CD8^+^ T cell may be affected during chronic HCV infections. IL-10 produced by macrophages, DC, regulatory T cells, and Th2 cells can suppress T cell function [200,201]. An increased secretion of IL-10 has been observed for various chronic viral infections, including HCV [202,203]. This impairment of T cell function, particularly that of CD4^+^ and CD8^+^ T cells, by an increased expression of IL-10 has also been supported by studies involving the LCMV model [204,205]. While allowing viral persistence, the presence of IL-10 in the liver could also be beneficial in regulating constantly activated T cells that could aggravate immunopathology and cause fibrosis of the liver [206].

Regulatory T cells (T_regs_) have an important role to play in the viral persistence in a chronic HCV infection. In recent years, studies have focused on the role of regulatory T cells (T_reg_) in HCV infections to determine if they influence viral persistence. In patients with chronic hepatitis C, the frequency of CD4^+^CD25^+^ T cells (T_R_ cells) is reported to be high [207], and these cells can suppress virus-specific CD8^+^ T cells via the action of immunosuppressive cytokines they secrete. Depletion of CD4^+^CD25^+^ T_reg_ cells from peripheral blood resulted in the recovery of proliferation and peptide-specific IFN-γ production by HCV-specific CD8^+^ T cells [208]. These reports initially used CD25 as a marker for identifying regulatory T cells, which is also expressed by activated T cells. T_regs_ now are more precisely defined by another marker, the forkhead/winged helix transcription factor 3 (Foxp3). Recent reports also support the premise that Foxp3^+^ T_regs_ are elevated during a chronic HCV infection and that the maintenance of these cells may contribute to HCV persistence in some patients [209]. In chronic HCV-infected livers, Foxp3^+^ T_reg_ cells as well as IL-10 secreting virus-specific CCR7^-^ CD8^+^ T_R_ cells have been identified [202,210]. Most reports hint towards an increased frequency of T_reg_ cells and a suppressive activity associated with chronic disease. Though they may attenuate HCV-specific T cell responses in the liver, their presence may also reduce the risks of hepatic injury as incurred by the presence of a sustained CTL response [211]. Therefore, in an HCV infection, T_regs_ may function to downregulate the tissue damaging response to infection in liver as well as promote the maintenance of HCV persistence.

## 6. Impact of Host–HCV Interactions on HCV Therapy

Until recently, available therapeutic options for HCV infection were limited to pegylated interferon (PEG-IFN) and Ribavirin for all genotypes with a sustained virologic response (SVR) achievable in a subset of treated HCV-infected individuals [212]. However, patients undergoing interferon-based treatment often experienced adverse side effects, including fatigue, headache, pyrexia, myalgia, insomnia, alopecia, arthralgia, anorexia, tinnitus, and depression [213]. There are four classes of direct acting antivirals (DAA) that are being used in different combinations for all HCV genotypes and that form the mainstay of anti-HCV therapy [214]. The various DAAs classified on the basis of the targeted nonstructural protein and genotype are listed in Table 1. In comparison to interferons, DAAs are safer and more efficacious with concomitant improvement in SVR and reduced treatment duration.

IL-1β induces the chronic activation of innate immune-mediated inflammation [215,216]. DAA pharmacotherapy has been shown to reduce the innate immune activation through reduced production of IL-1β as well as reduced phosphorylation of NFκβ. This translates to a reduced inflammation with a consequential reduction in liver fibrosis and damage. The reduction in the expression of CXCL10 and CXCL11, chemokines that recruit innate immune cells, is observed with DAA pharmacotherapy. Furthermore, DAA therapy is associated with a normalization of NK cell function [217]. The reduced secretion of these chemokines along with the normalization of NK cell function correlates with a reversal of dysregulated innate immunity leading to reestablishing homeostasis of the innate immune system [218]. Alao et al. [219] demonstrated that baseline ISGs (Interferon stimulated genes) were upregulated in DAA-cured HCV patients, suggesting a role for innate immunity in the clearance of HCV during DAA therapy. It is of note that HCV NS3/4A protease interferes with RIG-I and TLR3 signaling by cleaving MAVS and TRIF, two human proteins known to play a critical role in innate immune response [144,145]. However, it is unclear whether NS3/4A protease inhibitors clear the virus because of their direct antiviral effect or because of their ability to boost the antiviral innate immune response by preventing the hydrolysis of TRIF and MAVS. Martin et al. [220] suggested that DAA-mediated removal of HCV antigens could have contributed to a restoration of the proliferative capability of exhausted HCV-specific CD8^+^ T cells in the majority of patients with a sustained virologic response 12 weeks after cessation of treatment (SVR12). This is likely to improve the adaptive immunity in these patients but not to the same level of improvement observed with DAA-associated reestablishment of innate immunity homeostasis [221]. A DAA-mediated cure of HCV is associated with the normalization of innate immunity with a partial restoration of exhausted HCV-specific CD8^+^ T cells that express low levels of PD-1 [222].

DAA-mediated HCV clearance normalizes innate immunity in HCV-cured individuals but provides only a partial restoration of adaptive immunity due to high PD-1 and low CD127 expressions on restored HCV-specific CD8^+^ T cells. Additionally, the emergence of DAA-resistant HCV variants poses a significant threat to strategies geared towards reducing HCV transmission, particularly in high risk groups. Furthermore, the high cost of current DAAs, which are unaffordable in resource-limited nations with a high prevalence of HCV, is another compelling reason to intensify efforts to develop an affordable and effective HCV vaccine. As such, vaccination strategies that either provide sterilizing immunity or protective immunity against the development of viral persistence upon reinfection would be immensely beneficial particularly in high risk groups who are most likely to be reinfected with HCV [223].

The development of a robust early humoral immune response via neutralizing antibodies in the initial phase of an HCV infection is likely to result in the spontaneous clearance of a viral infection [224,225]. The early and robust development of neutralizing antibodies is a correlate of protective immunity against developing viral persistence in HCV-infected individuals. Additionally, a spontaneous resolution of acute HCV has been shown to induce memory T-cell-induced protective immunity [226,227,228]. However, this protective immunity is not absolute since it cannot prevent reinfection by HCV variants that did not induce the preexisting memory T cells [227,229].

Although there are HCV vaccines at different stages of development, there is no FDA-approved HCV vaccine. Law et al. [230] demonstrated that an HCV vaccine comprising envelope glycoproteins gpE1/gpE2 derived from a single isolate induced broad cross-neutralizing antibodies against all HCV genotypes with varying efficiency. It also induced T-cell-mediated responses. Swadling et al. [231] demonstrated that a human prophylactic T-cell-based HCV vaccine induced the production of both CD4^+^ and CD8^+^ T cells. This vector-based vaccine that encoded nonstructural proteins uses a replicative defective Simian adenoviral vector as a prime and modifies vaccina Ankara (MVA) as a booster. The results of these clinical studies will be available in the future.

(1) An HCV genomic variability with seven distinct genotypes with more than 65 subtypes which differ in nucleotide sequence, (2) a high error prone mutation rate of HCV with the capability to escape selection pressure by neutralizing antibodies and CD8^+^ T cells [232], (3) a high mutation rate occurring in the hypervariable region 1 of E2 along with the potential of HVR 1 to interfere with the binding of antibodies to E2 [233], (4) the cell to cell transmission of HCV constituting a considerable hindrance to developing B-cell-based HCV vaccines that induce broad cross-neutralizing antibodies since HCV could avoid the extracellular compartment [234], and (5) HCV in circulation binding to plasma lipoprotein to form an infectious hybrid lipoviral particle (LVP) that promotes viral persistence and a high infection by limiting the access of neutralizing antibodies to envelop glycoprotein [235,236] are factors that poses a significant challenge to developing an effective HCV vaccine.

Because reinfection following cure of HCV is a possibility, there is a need to intensify efforts in the research and development of safe and effective HCV vaccines that induce the generation of cross-neutralizing antibodies that target epitopes that are conserved among HCV genotypes and not associated with HCV escape. It should be effective against the diverse variants of HCV since there is over 30% of nucleotide sequence diversity among the genotypes [226,237]. Finally, an HCV vaccine that can generate cross-neutralizing antibodies and cell-mediated immune responses should be the goal, since the generation of vaccine immunogen-induced humoral immunity is inadequate in providing protection against chronic HCV infections.

## 7. Conclusions

HCV, a major cause of liver disease, is a global health problem that causes liver cirrhosis in a third of individuals with chronic HCV infections. A few of these individuals with chronic HCV may develop HCC. Host and viral factors play a role in host–viral interactions that could result in a spontaneous resolution of the acute infection or a progression to a chronic HCV infection. NK cells provide innate cellular immunity via the secretion of type II IFN and TNFα that inhibit viral replication via noncytolytic-dependent mechanisms as well as secrete perforin and granzyme that destroy infected cells via cytolytic-dependent mechanisms. An adaptive cellular response to HCV infection is mainly mediated by CD8+ T cells that clear the virus via both cytolytic and noncytolytic mechanisms. CD4^+^ T cells provide help to CD8^+^ T cell, APC, and B cells. A failure of cellular immunity correlates with an impaired control of HCV infection. The immunosuppressive action of induced regulatory T cells, an impaired antigen presentation by HCV-infected DC, HCV escape mutation, T cell exhaustion due to persistent HCV antigens, an impaired priming of T cells by DC and intrahepatic antigen presenting cells, and an induction of tolerogenic intrahepatic microenvironment are factors that promote the persistence of HCV infection. A considerable knowledge of the host and virus interaction in terms of factors that promote a resolution of the acute phase of an HCV infection and immune evasive strategies employed by HCV to maintain a persistence in the host is necessary. Despite the effectiveness with which DAAs act on various viral proteins such as NS5A, NS5B, and NS3/4 protease, HCV still remains evasive in some population. Because reinfection following the cure of HCV is a possibility, there is a need to develop an affordable and effective HCV vaccine. However, efforts to develop an HCV vaccine are hampered by viral factors such as HCV genomic diversity, the cell to cell spread of HCV, a high mutation rate, and the development of infectious lipoviral particles. Because the immune response to an HCV infection is protective, ongoing research to develop a safe and affordable vaccine will provide hope for millions of individuals at risk of HCV infection.

## Figures and Tables

**Table 1:** The four classes of direct acting antivirals (DAAs) that are being used in different combinations and that form the mainstay of anti-HCV therapy.

Class of DAA	DAA (Targeted Genotypes in Brackets)
NS3/4A Protease Inhibitors (PIs)	Glecaprevir (1–6) Voxilaprevir (1–6) Galexos (1) Grazoprevir (1, 3, 4) Sunvepra (1, 4)
Nucleoside and Nucleotide NS5B Polymerase Inhibitors	Sofosbuvir (1–4)
NS5A Inhibitors	Ombitasvir (1, 4) Pibrentasvir (1–6) Daclatasvir (3) Elbasvir (1, 4) Ombitasvir (1) Velpatasvir (1–6)
Non-Nucleoside NS5B Polymerase Inhibitors	Dasabuvir (1)
